# Supplementary material for: Is serotonin transporter brain binding associated with the cortisol awakening response? An independent non-replication
Source: PLoS One. 2023 Aug 31;18(8):e0290663. doi: 10.1371/journal.pone.0290663 (PMC10470919; doi:10.1371/journal.pone.0290663)
Supplement: S1 Table — (PDF) [file pone.0290663.s001.pdf]

S1 Table.

S1 Table. Average 5-HTT BP_{ND} and linear regression results for association between CAR and 5-HTT BP_{ND}.

ROI	Mean 5-HTT BP_{ND} ±SD	Estimate (β)	p-value
Amygdala	1.83 ± 0.32	-0.69	0.56
Anterior cingulate cortex (ACC)	0.64 ± 0.11	-0.64	0.11
Caudate nuclei	1.9 ± 0.32	-2.4	0.04
Hippocampus	0.73 ± 0.13	-0.32	0.51
Insula	0.91 ± 0.14	-0.8	0.13
Posterior cingulate cortex (PCC)	0.52 ± 0.1	-0.086	0.79
Putamen	2.3 ± 0.36	-2.3	0.08
Thalamus	2.7 ± 0.35	-1.6	0.19

All models are corrected for age and sex.
