# Supplementary material for: Is serotonin transporter brain binding associated with the cortisol awakening response? An independent non-replication
Source: PLoS One. 2023 Aug 31;18(8):e0290663. doi: 10.1371/journal.pone.0290663 (PMC10470919; doi:10.1371/journal.pone.0290663)
Supplement: S1 Appendix — Figures A-D. (PDF) [file pone.0290663.s002.pdf]

S1 Appendix

Figure (A-D).

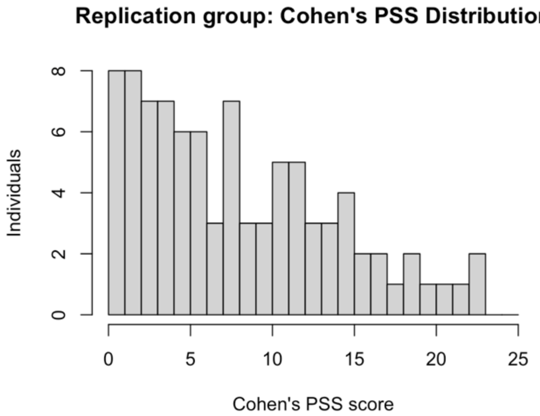

Figure A. Histogram illustrating distribution of Cohen's Perceived stress score in the replication group.

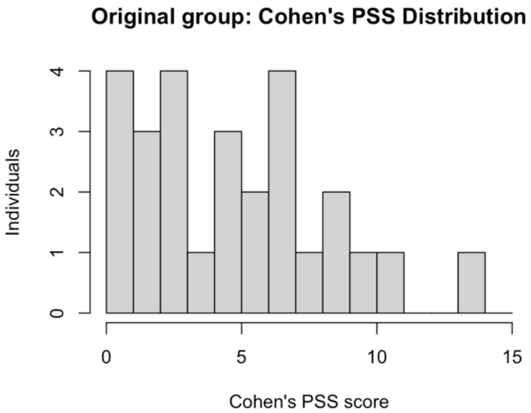

Figure B. Histogram illustrating distribution of Cohen's Perceived stress score in the original group.

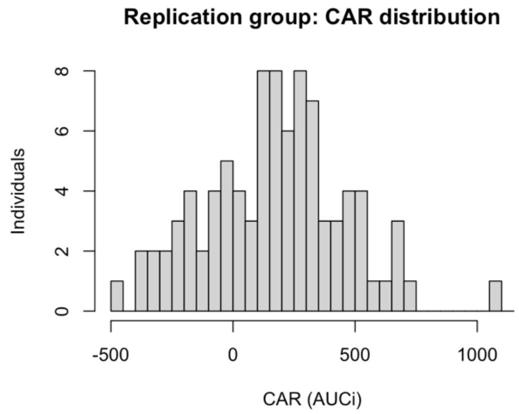

Figure C. Histogram illustrating distribution of CAR in the replication group.

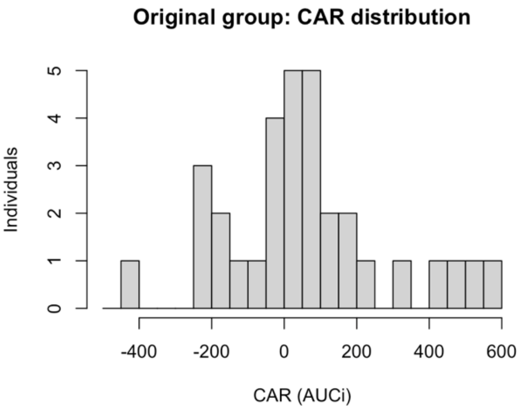

Figure D. Histogram illustrating distribution of CAR in the original group.
