# Supplementary material for: Is serotonin transporter brain binding associated with the cortisol awakening response? An independent non-replication
Source: PLoS One. 2023 Aug 31;18(8):e0290663. doi: 10.1371/journal.pone.0290663 (PMC10470919; doi:10.1371/journal.pone.0290663)
Supplement: S1 Text — (PDF) [file pone.0290663.s003.pdf]

ASPREDICTED

Wharton CREDIBILITY LAB UNIVERSITY of PENNSYLVANIA

Prefrontal 5-HTT and cortisol awakening response: a replication study (#80805)

Created: 11/22/2021 11:11 PM (PT)
Public: 05/09/2022 05:27 AM (PT)

Author(s)
Jonas Svensson (Neurobiology Research Unit, Denmark) - jonas.svensson@nru.dk
Vibe Frøkjær (Neurobiology Research Unit, Denmark) - vibe.frokjaer@nru.dk
Søren Vinther (Neurobiology Research Unit, Denmark) - soren.vinther@nru.dk

1) Have any data been collected for this study already?
It's complicated. We have already collected some data but explain in Question 8 why readers may consider this a valid pre-registration nevertheless.

2) What's the main question being asked or hypothesis being tested in this study?
This study will attempt to replicate the findings of a positive association between prefrontal serotonin transporter (5-HTT) availability and cortisol awakening response (CAR) reported in Frokjaer 2013.

3) Describe the key dependent variable(s) specifying how they will be measured.
The radioligand [11C]DASB binds specifically to the serotonin transporter, and this binding can be quantified using positron emission tomography (PET). The reported outcome measure will be non displaceable binding potential (BPND), un-corrected for partial volume effects. Quantification of radioligand binding will be done using MRTM2 with a time-activity curve from the cerebellum as reference.

4) How many and which conditions will participants be assigned to?
No conditions, healthy controls.

5) Specify exactly which analyses you will conduct to examine the main question/hypothesis.
CAR is based on five serial measurements of the rise in salivary cortisol over the first hour from awakening (0, 15, 30, 45 and 60 min). The area under curve from 0 to 60 min from awakening, with respect to increase from the baseline awakening cortisol value, AUC(i), will be calculated and used for the analysis. In case CAR is collected in shorter or longer intervals (based on selfreported saliva sampling timing, the AUCi will be normalized to 60 minutes).
93 subjects will be available for the replication study. In the original study an analysis of parametric images showed the largest effect to be approximately located within Brodmanns area 25. In the confirmatory analysis we will use FreeSurfer to segment the brain and use "Gyrus subcallosal" (which encompasses area 25) as the primary region of interest (ROI).
We will first confirm that a positive association, of a magnitude similar to what was reported in the original analysis, is seen in the 32 original subjects when the Gyrus subcallosal ROI is used. If so, we will proceed according to analysis plan 1). If not, we will proceed according to analysis plan 2).
Analysis plan 1) for the 93 subjects not included in the original analysis, we will use a linear regression model to test the association between CAR AUC(i) and Gyrus subcallosal BPND, while adjusting for age, sex, and type of PET system used. The test will be one sided, expecting a positive correlation, as was reported in the original paper.
In addition, a replication Bayes factor approach will be adopted (Ly, 2019), where the evidence in favor of a successful replication of the original dataset (n=32) (H1) against the hypothesis of no association (H0), will be assessed in the replication dataset. The prior over beta-CAR in the original dataset will be Gaussian distribution, centered around 0 with an SD of 10.
Analysis plan 2) if the Gyrus subcallosal ROI doesn't reproduce the original results, we will instead use the exact same delineation of brain regions as was done originally (i.e., the prefrontal cortex region from the NRU template atlas (Svarer, 2004)). The same statistical model will be applied as described above. Replication Bayes factor as described above will also be assessed.
Regardless of whether plan 1 or 2 is used, we will also test if an association between CAR and BPnd depends on sex
Hence, in the confirmatory analysis, one test for statistical significance will be performed. The test will be one sided and alpha will be set to 0.05. A BF > 3 will be considered a successful replication.

6) Describe exactly how outliers will be defined and handled, and your precise rule(s) for excluding observations.
Two subjects have anomalies described in the collection of CAR data (issues at test collection; and ongoing medication known to interfere with cortisol measurements) and have therefore been excluded. In the PET data, regions where the modeling have failed (eg negative BPND values) will be excluded.

7) How many observations will be collected or what will determine sample size? No need to justify decision, but be precise about exactly how the number will be determined.
This is an analysis in an existing database, the sample sized is therefore fixed (see under pt 5).

8) Anything else you would like to pre-register? (e.g., secondary analyses, variables collected for exploratory purposes, unusual analyses planned?)
The data is already collected and stored in a database. However, at the time of preregistration no analysis of the PET data has been performed. We therefore consider this a confirmatory analysis.
We plan to perform several exploratory analyses, we will:
1) Dichotomize the CAR in "blunted" and "non-blunted". CAR will be defined as non-blunted if 1) there is a positive AUCi and 2) 50% minimum increase of

AUC(i) from baseline, calculated as ((cortisol peak – cortisol baseline)/cortisol baseline)� 100%. The remaining CAR measures will be classified as blunted.
2) explore other brain regions, e.g., raphe nuclei, hippocampus, paraventricular nucleus, dorsolateral prefrontal cortex.
3) explore the hypothesis that individuals with significant childhood trauma have a long-term blunted CAR. The "Parental bonding subjective ratings for both mother and father relation scale" will be used for this purpose.
4) attempt to include other information into the statistical model to explore if the association between CAR and [11C]DASB binding depends on several variables;. this will include data on stress (Stressful Life Experiences scale, Perceived Stress Scale), sleep (Pittsburgh sleep quality index), use of oral contraception (in a within premenopausal women only subgroup), Personality factor neuroticism score, season, 5HTTLPR gene variant status (high vs low expressing).
