# Supplementary material for: Is serotonin transporter brain binding associated with the cortisol awakening response? An independent non-replication
Source: PLoS One. 2023 Aug 31;18(8):e0290663. doi: 10.1371/journal.pone.0290663 (PMC10470919; doi:10.1371/journal.pone.0290663)
Supplement: Attachment — Submitted filename: Response to reviewers.docx [file pone.0290663.s004.docx]

**Response to Reviewer comments**

We thank the reviewers for valuable comments on our manuscript *Is serotonin transporter brain binding associated with the cortisol awakening response? An independent non-replication* (MS Number: PONE-D-23-06771). We have amended the text according to the reviewer’s comments and believe that the manuscript has improved as a result.

Below follows a point-by-point response, first to the comments by, the academic editor, thereafter to the comments by the reviewers. Our responses are written (in roman) to each point raised (*in italics*).

Points raised by the academic editor

*1. Please ensure that your manuscript meets PLOS ONE's style requirements, including those for file naming. The PLOS ONE style templates can be found at*

*https://journals.plos.org/plosone/s/file?id=wjVg/PLOSOne_formatting_sample_main_body.pdf and*

*https://journals.plos.org/plosone/s/file?id=ba62/PLOSOne_formatting_sample_title_authors_affiliations.pdf.*

We have updated the manuscript to meet PLOS ONE’s style requirements using the recommended style templates. See the ‘Manuscript’ file.

*2. In the Methods section and the online submission form, please provide additional information about the patient records used in your retrospective study. Specifically, please ensure that you have discussed whether all data were fully anonymized before you accessed them and/or whether the IRB or ethics committee waived the requirement for informed consent. If patients provided informed written consent to have data from their medical records used in research, please include this information.*

The participants in both the original and replication study were healthy controls in previous clinical studies accessible upon application within the Cimbi database structure. We have updated this information in the methods section, see paragraph 1.

*3. Thank you for stating in your Funding Statement:*

*"Database infrastructure was supported by The Research Council of Rigshospitalet, grantID A6594 and The Lundbeck Foundation, grantIDs R279-2018-1145 and R90-A7722. JC was supported by "Ivan Nielsens fond for personer med specielle sindslidelser", grantID 07018017 and the Independent Research Fund Denmark, grantID 7025-00111B. JS was supported by a grant from Capital Region's Research Foundation for Health Research, grantID R162-A7167. SVL was supported by the Independent Research Fund Denmark, grantID 0134-00278B and 7025-00111B."*

*Please provide an amended statement that declares *all* the funding or sources of support (whether external or internal to your organization) received during this study, as detailed online in our guide for authors at* [*http://journals.plos.org/plosone/s/submit-now*](http://journals.plos.org/plosone/s/submit-now)*. Please also include the statement "There was no additional external funding received for this study." in your updated Funding Statement.*

*Please include your amended Funding Statement within your cover letter. We will change the online submission form on your behalf.*

Please see the updated funding statement in ‘Cover letter’.

*4. We note that the grant information you provided in the 'Funding Information' and 'Financial Disclosure' sections do not match.*

*When you resubmit, please ensure that you provide the correct grant numbers for the awards you received for your study in the 'Funding Information' section.*

We have ensured that the correct grant information is provided in the ‘funding information’ section in the submission system.

*5. Thank you for stating the following in the Competing Interests section:*

*"I have read the journal's policy and the authors of this manuscript have the following competing interests: VGF has received honorarium as a consultant for Sage Therapeutics and lectures for Lundbeck Pharma A/S and Janssen-Cilag A/S."*

*We note that one or more of the authors are employed by a commercial company: Sage Therapeutics and lectures for Lundbeck Pharma A/S and Janssen-Cilag A/S*

a. *Please provide an amended Funding Statement declaring this commercial affiliation, as well as a statement regarding the Role of Funders in your study. If the funding organization did not play a role in the study design, data collection and analysis, decision to publish, or preparation of the manuscript and only provided financial support in the form of authors' salaries and/or research materials, please review your statements relating to the author contributions, and ensure you have specifically and accurately indicated the role(s) that these authors had in your study. You can update author roles in the Author Contributions section of the online submission form.*

*Please also include the following statement within your amended Funding Statement.*

*"The funder provided support in the form of salaries for authors [insert relevant initials], but did not have any additional role in the study design, data collection and analysis, decision to publish, or preparation of the manuscript. The specific roles of these authors are articulated in the 'author contributions' section."*

*If your commercial affiliation did play a role in your study, please state and explain this role within your updated Funding Statement.*

b. *Please also provide an updated Competing Interests Statement declaring this commercial affiliation along with any other relevant declarations relating to employment, consultancy, patents, products in development, or marketed products, etc.*

*Within your Competing Interests Statement, please confirm that this commercial affiliation does not alter your adherence to all PLOS ONE policies on sharing data and materials by including the following statement: "This does not alter our adherence to PLOS ONE policies on sharing data and materials." (as detailed online in our guide for authors* [*http://journals.plos.org/plosone/s/competing-interests*](http://journals.plos.org/plosone/s/competing-interests)*). If this adherence statement is not accurate and there are restrictions on sharing of data and/or materials, please state these. Please note that we cannot proceed with consideration of your article until this information has been declared.*

*Please include both an updated Funding Statement and Competing Interests Statement in your cover letter. We will change the online submission form on your behalf.*

Thank you for your guidance on how to update the competing interests and funding statement to meet PLOS ONE requirements. See ‘Cover letter’ for updated sections. We would like to make it clear that none of the authors have any employment or affiliation with any commercial organization. We have updated the funding statement stating the role of the funders.

*While revising your submission, please upload your figure files to the Preflight Analysis and Conversion Engine (PACE) digital diagnostic tool, https://pacev2.apexcovantage.com/. PACE helps ensure that figures meet PLOS requirements. To use PACE, you must first register as a user. Registration is free. Then, login and navigate to the UPLOAD tab, where you will find detailed instructions on how to use the tool. If you encounter any issues or have any questions when using PACE, please email PLOS at figures@plos.org. Please note that Supporting Information files do not need this step.*

We have updated the figures to fit your requirements using the PACE tool. The figure file is inserted in the manuscript and uploaded as a separate file in the submission system.

**Points raised by the reviwers**

***Reviewer #1:*** *The authors report failing to replicate a previously reported finding from this group and awakening cortisol response correlates positively with PFC 5-HTT binding. There are several aspects of this study that seem glossed over.*

*1) First the two study samples were scanned with different PET scanner and the 5-HTT binding levels differ significantly. The cortisol levels and reported perceived stress levels also differ between the two samples. Even in the first sample the correlations was present in only one of the two brain regions examined. So there are many ways by which the study samples differ including on the two measures that they are seeking a correlation for in this study.*

There are indeed differences in key variables between the original sample and the replication sample. We have now expanded on this discussion in the manuscript (see the ‘Non-replication’ paragraph in the discussion section). The camera system used in the replication sample has a higher resolution, which, through reduced partial volumes effects, will lead to different binding estimates than lower-resolution systems. This is a well-described phenomenon, and while it can make it difficult to directly translate binding estimates between experiments on different systems, the rank order between subjects, and thus any association with other measurements (e.g., CAR data) should be the same regardless of camera.

Therefore, given a robust and generalizable underlying association between the examined variables, we would expect it to be detected regardless of these differences

*2) They have an odd idea that high transporter binding means lower serotonin release but in fact it is intra-synaptic cortisol that regulates HTT internalization and so when there is less serotonin release this increase internalization of 5-HTT and reduces the number of transporters.*

Thank you for highlighting this section of the text which indeed is speculative and not something that our data can directly inform on. We have now revised the manuscript accordingly (Introduction, second paragraph).

*3) There are technical issues. They do not employ an input function and so factors like brain blood flow or variable nonspecific binding could add noise and they cannot detect that.*

We agree that the use of reference tissue modeling for quantification in neuro PET comes with certain limitations and can contribute to noise. A sentence regarding this has been added to the discussion section (paragraph under the header “Methodological considerations”). For [11C]DASB, the radioligand used in this study, reference tissue modeling has been common practice for more than two decades[1]. Notably, the same method is applied in the replication study as in the original study.

*4) They could present data on more ROIs from the original sample as well as the replication sample and see how widespread or narrow the original finding really was and the same for the replication sample.*

In the original study, the association between CAR and [11C]DASB binding was reported in the prefrontal cortex and the anterior cingulate cortex (ACC). In both regions, a positive association was observed, but statistically significant only in the prefrontal cortex. Previously we had not reported on ACC in the replication sample but have now added this analysis to the manuscript. The association was negative and not significant (see methods section under “Regions of interest” and results section under “Association between 5-HTT binding and CAR”).

The primary objective of the current study is to attempt a replication of the significant finding in the original study. Although we see value in a larger exploratory analysis beyond this, such an approach comes with a risk of new spurious findings, undermining the purpose of the replication attempt. Beyond adding ACC to the analysis of the replication sample we are hesitant to add further analyses to this study.

*5) Finally they can put their CAR response in perspective by looking at other PET studies of serotonin markers and cortisol like Steinberg et al and Bartlett et al from Columbia group.*

The work on this topic by the Columbia group is indeed central and the lack of incorporation of this in the manuscript was noted by both reviewers. We have now added central references in the appropriate sections of the manuscript (see discussion section under heading 3 and 4.)

***Reviewer #2:***  *This manuscript attempts to replicate the authors' prior findings of a relationship between serotonin transporter or 5HTT measured using [11C]DASB PET binding and cortisol awakening response (CAR), an index of HPA axis stress hormone dynamics, in healthy adults. The authors observed that prefrontal 5-HTT binding is positively associated with CAR. The authors were not able to replicate their prior findings in a sample of 90 healthy adults.This is an important research question to understand the role of HPA axis dynamics in serotonin availability. Replicating a prior finding is also very important given the limited reproducibility of findings. However, there were several concerns about the manuscript.*

*1) The manuscript was not clear on the original sample size and the nature of the sample. This manuscript needs to be evaluated without the need to go to the published findings and some of the study design details of the original sample are important to present here. This is especially important as it seemed from the discussion that the original sample was a healthy sample.*

Thank you for pointing this out, we have now clarified the information about the cohort studied in the original study. See updated table 1 and methods section, first paragraph.

*2) The definition of blunted HPA axis activity by using <50% increase in CAR is not justified. The authors has already analyzed this variable continuously. Without a rationale that is well justified, this definition is arbitrary.*

Thank you, upon reconsideration we agree with this point. This analysis has now been removed from the manuscript.

*3) The sample sizes in the table comparing the samples need to be presented. There were differences between the samples on BMI but the authors did not control for BMI.*

The table is now updated. We have run the analysis with BMI as a covariate. This did not change the results notably. This analysis is reported in the last paragraph of the results section.

*4) The manuscript does not acknowledge work from the Columbia group (Dr. John Mann) where they used this ligand in depressed samples.*

The work on this topic by the Columbia group is indeed central and the lack of incorporation of this in the manuscript was noted by both reviewers. We have now added central references in the discussion section under heading 3 and 4.)

References:

1.	Ichise M, Liow JS, Lu JQ, Takano A, Model K, Toyama H, et al. Linearized reference tissue parametric imaging methods: application to [11C]DASB positron emission tomography studies of the serotonin transporter in human brain. J Cereb Blood Flow Metab. 2003;23(9):1096-112. Epub 2003/09/16. doi: 10.1097/01.WCB.0000085441.37552.CA. PubMed PMID: 12973026.
