# Supplementary material for: Is serotonin transporter brain binding associated with the cortisol awakening response? An independent non-replication
Source: PLoS One. 2023 Aug 31;18(8):e0290663. doi: 10.1371/journal.pone.0290663 (PMC10470919; doi:10.1371/journal.pone.0290663)
Supplement: Attachment — Submitted filename: Response to reviewers.docx [file pone.0290663.s005.docx]

**Response to Reviewer comments**

We thank the reviewers for valuable comments on our manuscript *Is serotonin transporter brain binding associated with the cortisol awakening response? An independent non-replication* (MS Number: PONE-D-23-06771R1). We have amended the manuscript accordingly to the reviewer’s comments and believe that the manuscript has further improved.

Below follows a point-by-point response to the comments by the reviewers. Our responses are written (in roman) to each point raised (*in italics*).

**Points raised by reviewer #2**

***Reviewer*** *point 1: However, one remaining concern is the differences between the original sample and replication samples on perceived stress and CAR. There might be some outliers on these measures that are driving these differences. More details about this is needed.*

We agree that the differences between the original and replication sample on central parameters is an important limitation for the replication study. In order to assess whether the observed difference could be driven by the presence of outliers in our data we have performed the following analyses:

1) For both CAR and perceived stress score we are now reporting both mean and median values in the results section. No large differences indicative of skewed distributions were observed.

2) Histograms for both variables have been attached as supplementary figures. No clear outliers can be readily identified in these figures.

3) The differences between groups remain significant when applying the Wilcoxon signed rank test, which is less sensitive to outliers compared with the t-test (p=0.03 and p=0.02 for the difference between CAR and Cohen’s PSS respectively).

We have attempted to outline possible explanations for the difference between the original and replication group in CAR and Cohen’s PSS:

“There are several possible explanations for why we did not replicate our earlier finding. First, the present study was performed in a group of primarily young women, while participants in the original study was mostly men of a wider age range. CAR dynamics have been shown to differ between men and women [1]. This could explain the differences in average CAR between the two groups and affect an association with 5-HTT, especially since HPA-axis activity is affected by hormonal status in women [2]. However, the replication analysis was controlled for both age and sex, and also an interaction analysis did not indicate that the association between CAR and 5-HTT was dependent on sex. Therefore, we consider this explanation less likely.”

And

“The recruitment of the control group was done to match a study group using recreational drugs such as MDMA, which could contribute to differences between the participants in the original- and replication study. For instance, the two groups differed significantly on average Cohen’s perceived stress scores, where the replication group scored higher and had a lower average BMI. In a sensitivity analysis, however, adding these variables as covariates did not change the association’s direction, effect size, or significance level. Histograms illustrating the frequency distribution of CAR and Cohen’s PSS for the two study groups, did not reveal any clear outliers driving the between-group differences for these parameters (see Supporting information, Figure A-D in S1 Appendix.)

***Reviewer*** *point 2: In addition, given the differences between the samples, the authors need to tone down the replication perspective of this work.*

We agree with the reviewer that this effort to replicate an earlier finding is not strictly a direct replication of the original study due to differences in key variables. We are now referring to this work as a conceptual replication in the manuscript:

Given the above differences between the original sample and the replication sample this study cannot be considered a *direct* replication attempt, but can be viewed as a conceptual replication[3, 4].

We have toned down the wording regarding replication throughout the manuscript:

- We omitted ‘replicate’ from this sentence in introduction section:

“Here we aim to test our previous findings of a positive association between prefrontal 5-HTT binding and CAR in a larger, independent dataset, with a higher proportion of women and a PET-system with higher resolution compared with the original study.”

- We replaced ‘replicate’ with ‘find’ in this sentence of the methods section:

“In the analysis of the replication group, we did not find a significant association between prefrontal 5-HTT binding and CAR, adjusting for age and sex (β= -0.28, p=0.26) (Fig 1).”

- In the discussion we previously propose that the non-replication could be explained by the original finding being a type I error. However, such statement is too strong given that the present work cannot qualify as a direct replication. Instead we now write:

“Fourth, another explanation for the non-significant finding in this replication effort is that there is no direct coupling between prefrontal 5-HTT binding and cortisol dynamics, or that it is not possible to reliably detect such coupling using [^11^C]DASB PET and CAR.”

- In the conclusion we changed the wording from ‘independent replication study’ to ‘replication attempt’ in the following sentence:

“In this replication attempt, we could not confirm a positive association between CAR and prefrontal 5-HTT BP_ND_ in healthy adults.”

***Reviewer*** *point 3: This is not strictly a replication and it is a missed opportunity not to look at other brain regions given the larger sample of the "replication sample". The original sample is small and could have resulted in a spurious association that cannot be replicated.*

We have now as supplementary material provided additional exploratory analyses in 8 brain regions with a relatively high density of serotonin transporters (Anterior cingulate cortex, putamen, caudate nuclei, thalamus, insula, hippocampus, amygdala, posterior cingulate cortex). All observed associations were negative (i.e., in the opposite direction compared with the original finding), this is expected given the high correlation between brain regions for the in vivo concentration of most examined proteins related to the serotonin system [5]. To note, the analysis showed a p-value of 0.04 in one region (caudate nucleus), though this result would not survive an adjustment for multiple comparisons, it could warrant future attention.

We have added this to the ’Perspectives’ paragraph of the discussion section:

“Our exploratory analysis of the independent dataset showed negative associations between 5-HTT binding and CAR in all examined brain regions, with the strongest association in the caudate nuclei (β = -2.4, p=0.04). Given that this was an exploratory analysis on a relatively large number of ROIs, without correction for multiple comparisons, this result should be interpreted with utmost caution but could lend some support for future studies focusing on regional specificity.”

1.	Nasser A, Ozenne B, Hogsted ES, Jensen PS, Frokjaer VG. Reliability of three versus five saliva sampling times for assessing the cortisol awakening response. Psychoneuroendocrinology. 2022;147:105950. Epub 2022/10/23. doi: 10.1016/j.psyneuen.2022.105950. PubMed PMID: 36272363.

2.	Gervasio J, Zheng S, Skrotzki C, Pachete A. The effect of oral contraceptive use on cortisol reactivity to the Trier Social Stress Test: A meta-analysis. Psychoneuroendocrinology. 2022;136:105626. Epub 2021/12/19. doi: 10.1016/j.psyneuen.2021.105626. PubMed PMID: 34922094.

3.	Zwaan RA, Etz A, Lucas RE, Donnellan MB. Making replication mainstream. Behav Brain Sci. 2017;41:e120. Epub 2017/10/27. doi: 10.1017/S0140525X17001972. PubMed PMID: 29065933.

4.	Nosek BA, Errington TM. Making sense of replications. Elife. 2017;6. Epub 2017/01/20. doi: 10.7554/eLife.23383. PubMed PMID: 28100398; PubMed Central PMCID: PMCPMC5245957.

5.	Beliveau V, Ozenne B, Strother S, Greve DN, Svarer C, Knudsen GM, et al. The structure of the serotonin system: A PET imaging study. Neuroimage. 2020;205:116240. Epub 2019/10/11. doi: 10.1016/j.neuroimage.2019.116240. PubMed PMID: 31600591; PubMed Central PMCID: PMCPMC6951807.
